# Scalable Process for High-Yield Production of *Pf*CyRPA Using Insect Cells for Inclusion in a Malaria Virosome-Based Vaccine Candidate

**DOI:** 10.3389/fbioe.2022.879078

**Published:** 2022-05-20

**Authors:** Bárbara Fernandes, Marcos Sousa, Rute Castro, Anja Schäfer, Julia Hauser, Kai Schulze, Mario Amacker, Marco Tamborrini, Gerd Pluschke, Paula M Alves, Sylvain Fleury, António Roldão

**Affiliations:** ^1^ iBET-Instituto de Biologia Experimental e Tecnológica, Oeiras, Portugal; ^2^ ITQB NOVA-Instituto de Tecnologia Química e Biológica António Xavier, Universidade Nova de Lisboa, Oeiras, Portugal; ^3^ Swiss Tropical and Public Health Institute, Basel, Switzerland; ^4^ University of Basel, Basel, Switzerland; ^5^ Helmhotz Center for Infecion Research, Braunschweig, Germany; ^6^ Mymetics SA, Épalinges, Switzerland; ^7^ Department of Pulmonary Medicine, Bern University Hospital, University of Bern, Bern, Switzerland

**Keywords:** insect cells, BEVS, bioprocess engineering, malaria vaccine, *Pf*CyRPA

## Abstract

*Plasmodium falciparum* cysteine-rich protective antigen (*Pf*CyRPA) has been identified as a promising blood-stage candidate antigen to include in a broadly cross-reactive malaria vaccine. In the last couple of decades, substantial effort has been committed to the development of scalable cost-effective, robust, and high-yield *Pf*CyRPA production processes. Despite insect cells being a suitable expression system due to their track record for protein production (including vaccine antigens), these are yet to be explored to produce this antigen. In this study, different insect cell lines, culture conditions (baculovirus infection strategy, supplementation schemes, culture temperature modulation), and purification strategies (affinity tags) were explored aiming to develop a scalable, high-yield, and high-quality *Pf*CyRPA for inclusion in a virosome-based malaria vaccine candidate. Supplements with antioxidants improved *Pf*CyRPA volumetric titers by 50% when added at the time of infection. In addition, from three different affinity tags (6x-His, 4x-His, and C-tag) evaluated, the 4x-His affinity tag was the one leading to the highest *Pf*CyRPA purification recovery yields (61%) and production yield (26 mg/L vs. 21 mg/L and 13 mg/L for 6x-His and C-tag, respectively). Noteworthy, *Pf*CyRPA expressed using High Five cells did not show differences in protein quality or stability when compared to its human HEK293 cell counterpart. When formulated in a lipid-based virosome nanoparticle, immunized rabbits developed functional anti-*Pf*CyRPA antibodies that impeded the multiplication of *P. falciparum in vitro*. This work demonstrates the potential of using IC-BEVS as a qualified platform to produce functional recombinant *Pf*CyRPA protein with the added benefit of being a non-human expression system with short bioprocessing times and high expression levels.

## 1 Introduction

Malaria is responsible for more than 627000 deaths per year, thus urging the need to develop a highly effective vaccine to control and eradicate this disease (World Health Organization, 2021). Recently, RTS,S/AS01 vaccine (Mosquirix^TM^) against *Plasmodium falciparum* was the first malaria vaccine to get the WHO recommendation for wider use. However, its efficacy is still modest; thus, the development of an effective second-generation malaria vaccine able to provide broad coverage against parasite infection is a public health priority (Arora et al., 2021).

A highly effective malaria subunit vaccine needs to incorporate multiple conserved antigens for optimal long-term protection, and ideally having an essential protein–protein interaction devoid of alternative pathways. The *Pf* cysteine-rich protective antigen (*Pf*CyRPA) fulfills these criteria and has emerged as a promising blood-stage candidate antigen with very limited genetic diversity that could elicit protective antibodies cross-reacting toward various *Pf* geographical strains (Dreyer et al., 2012; Favuzza et al., 2016).

A suitable manufacturing platform for the production of biologic products requires a cost-effective, scalable, and robust expression system. Recombinant *Pf*CyRPA has been produced in bacteria (Dreyer et al., 2012) and human HEK293 cells (Favuzza et al., 2016). Insect cells feature several advantages over mammalian and human cells, including ease of culture and high production yields of antigens with human-like folding and post-translational modifications, in short time frames and at low cost (Jarvis, 2003; Ahn et al., 2008). Different FDA-approved vaccines for human use produced in the insect cell baculovirus expression vector system (IC-BEVS), such as Cervarix® against human papillomavirus, Flublok against influenza virus, and, more recently, Nuvaxovid against SARS-CoV-2, which underlines the high potential and versatility of this platform.

Several optimization strategies in insect cells and IC-BEVS have been attempted to cope with manufacturing pressure (Correia et al., 2020; Fernandes et al., 2020).

Screening of the most suitable cell line and fine-tuning infection strategy (e.g., multiplicity of infection, cell concentration at infection, and time of harvest) and process parameters (e.g., culture temperature) is essential to optimize protein expression in IC-BEVS (Aucoin et al., 2007; Sequeira et al., 2018; Correia et al., 2020; Fernandes et al., 2020). The increased interest in this expression system motivated the analysis of cell culture parameters and media components, resulting in the identification of specific supplements to modulate key cellular pathways influencing productivity (Monteiro et al., 2016). In addition, producing recombinant antigen proteins with the proper affinity tag is of utmost interest to facilitate the purification process (Jin et al., 2017) and reduce process costs.

Improving expression yield in insect cells can further revamp the interest and applicability of this expression system for vaccine manufacturing. This study describes, for the first time, the use of IC-BEVS for the expression of *Pf*CyRPA protein and its inclusion in a virosome-based vaccine candidate. Different upstream and downstream process optimization strategies such as screening for best-performing cell line and affinity tag, supplementation regimens, and the modulation of culture temperature were combined to further improve this expression platform to produce highly immunogenic *Pf*CyRPA.

## 2 Materials and Methods

### 2.1 Cell Lines and Culture Media

Insect *Sf-9* (Invitrogen) and High Five (Invitrogen) cells were routinely subcultured at 0.4–1 × 10^6^ cell/mL every 3–4 days when cell density reached 2–3x10^6^cell/mL, as described elsewhere (Fernandes et al., 2020). Insect-XPRESS^TM^ (Sartorius) and Sf-900^TM^ II SFM (Thermo Fisher Scientific) media were used to culture *Sf-9* and High Five cells, respectively. Human HEK293-E6 cells (NRC) (Durocher, 2002) were routinely subcultured to 0.5–0.6 × 10^6^ cells/mL every 3–4 days when cell density reached 2–3 × 10^6^ cells/mL in 125- or 500-mL shake flasks (20% working volume, w/v) in an Innova 44R incubator (orbital motion diameter of 2.54 cm Eppendorf) at 37°C with 5% CO_2_ and stirring rates of 75 or 90 rpm. FreeStyle F17 (Thermo Fisher Scientific) media, supplemented with 4 mM GlutaMAX™ (Thermo Fisher Scientific) and 0.1% of Pluronic™ F-68 (Life Technologies), and 25 µg/mL of Geneticin were used to culture the HEK293 cells.

### 2.2 Expression Vectors

For expression in HEK293 cells, the *Pf*CyRPA nucleotide sequence (Favuzza et al., 2017) was synthetically synthesized with the bee venom melittin (BVM) signal sequence to allow *Pf*CyRPA secretion into the culture medium, and a C-terminal tag (His_6_-tag) to allow purification, and cloned into the pTT5 vector, resulting in the pTT5-hCyRPA-6His plasmid. For expression in insect cells, the *Pf*CyRPA nucleotide sequence (Favuzza et al., 2017) was synthetically synthesized with the BVM signal sequence and a C-terminal tag (His_6_-tag, His_4_-tag, or C-tag) for purification, and cloned into the pOET3 vector, resulting in three different expression plasmids: pOET3-iCyRPA-His_6_, pOET3-iCyRPA-His_4_, and pOET3-iCyRPA-Ctag. All plasmids were synthetically synthesized by GenScript.

### 2.3 Baculovirus Generation

Recombinant baculovirus (rBac) containing pOET3-iCyRPA-His_6_, pOET3-iCyRPA-His_4_, or pOET3-iCyRPA-Ctag plasmids, from now on named “rBac-CyRPA-His6,” “rBac-CyRPA-His4,” and “rBac-CyRPA-Ctag,” respectively, were generated using the flashback ULTRA^TM^ system (Oxford Expression Technologies) in accordance with manufacturer’s instruction. The amplification of baculovirus stocks was performed as described elsewhere (Vieira et al., 2005). Briefly, *Sf-9* cells were infected at a cell concentration of 1 × 10^6^ cells/mL with 0.01–0.1 infectious baculovirus per cell (pfu/cell). When cell viability reached 80–85%, cultures were harvested and centrifuged at ×200g for 10 min at 4°C. The pellet was discarded, and the supernatant was centrifuged at ×2000g for 20 min at 4°C. The resulting supernatant was stored at 4°C until further use.

### 2.4 Production of *Pf*CyRPA Protein

#### 2.4.1 Insect Cells as Host

The production of *Pf*CyRPA using *Sf-9* and High Five cells was performed in shake flasks (SF; 500 mL, 10% w/v) and 2-L stirred tank bioreactors (STB). The cells were seeded at 0.6–0.3 × 10^6^ cells/mL and infected with rBac containing pOET3-iCyRPA-His_6_, pOET3-iCyRPA-His_4_, or pOET3-iCyRPA-Ctag plasmid at different cell concentrations at the time of infection (CCI, 1 × 10^6^ and 2 × 10^6^) and MOI (0.1 and 1 pfu/cell).

Bioreactor cultures in the batch mode were performed in a computer-controlled BIOSTAT^®^ DCU3 2 L vessel (Sartorius) equipped with two Rushton impellers and a ring sparger for gas supply. The pH was monitored (not controlled) along with culture time. The partial pressure of oxygen (pO_2_) was set at 30% of air saturation and was maintained by varying the agitation rate (70–250 rpm), and the percentage of O_2_ in the gas mixture (0–100%). The gas flow rate was set to 0.01 vessel volumes per minute (vvm). The temperature was set at 27°C, and the working volume was 2 L.

In the design of experiments (DoE) study (see Section 3.4), culture supplements known to enhance virus and recombinant protein production in IC-BEVS were tested (Table 1). The concentration of supplements was set in accordance with the manufacturer’s instructions and previously in-house developed work (Carinhas et al., 2010; Monteiro et al., 2016; Sequeira et al., 2018) and prepared using Insect-XPRESS™ (Sartorius). Culture supplementations and temperature shifts (to either 21, 31.5, or 36°C) were performed at the time of infection.

**TABLE 1:** List of culture medium supplements.

Supplement	Abbreviation	Stock concentration	Concentration added^a^	Supplier	References
Antioxidants	AOx	×1000	×1	Sigma	A1345
Polyamines	Pol	×100	×1	Sigma	G1404
Lipids	Lip	×100	×1	Gibco	11905–031
Disodium α-ketoglutarate	α-k	—	12 mM	Sial	K-3752

a Concentration of supplements added at time of infection.

#### 2.4 1 Human Cells as Host

The production of *Pf*CyRPA in human HEK293 cells was performed in SF (500 mL, 20% w/v) and 2-L STBs. The cells were seeded at 0.4 × 10^6^ cells/mL and transfected at a cell concentration of approximately 1.6 × 10^6^ cells/mL with the pTT5-hCyRPA-6His plasmid using polyethyleneimine (PEI, Polysciences) cationic polymer at a ratio of 1.5 mg PEI/1 mg plasmid DNA (pDNA) prepared in 10% (v/v) of total culture volume. Briefly, the PEI was slowly added in a dropwise manner to the pDNA medium mix, incubated for 8 min at room temperature (RT), and then the mix was added to the culture.

Bioreactor cultures in the batch mode were performed in a computer-controlled BIOSTAT^®^ DCU3 2 L vessel (Sartorius) equipped with two Rushton impellers and a ring sparger for gas supply. pO_2_ was set at 40% of air saturation and was maintained by varying the agitation rate (90–230 rpm) and the percentage of O_2_ in the gas mixture (0–100%). The gas flow rate was set to 0.01 vvm. The pH was set to 7.4 and controlled by CO_2_ and base addition (NaHCO_3_), and the temperature was kept at 37°C for a working volume of 2 L.

### 2.5 Purification of *Pf*CyRPA Protein

Purification of *Pf*CyRPA was carried out on an ÄKTA Explorer 100 System (Cytiva). Cell culture bulk was harvested, filtered through 0.45- and 0.22-μm Sartopore 2 Midicap Filter Cartridges 10 (Sartorius), and concentrated with a Sartocon disposable PES membrane 2 × 0.1 m^2^, 10 kDa (Sartorius). The concentrated sample was filtered through a Nalgene cup of 0.2 μm (Thermo Scientific). *Pf*CyRPA His_6_- and His_4_-tagged proteins were purified by immobilized metal ion affinity chromatography on a Histrap HP column (5 mL volume; Cytiva), while *Pf*CyRPA C-tag tagged protein was purified on a CaptureSelect™ C-tagaffinity matrix chromatography column (5 mL volume; Thermo Fisher). Column eluates were concentrated using an AmiconUltra 15 Centrifugal Filter Unit 10 kDa (Merck Millipore), filtered through 0.2 μm, and applied to a HiLoad 26/60 Superdex 75 gel permeation column (GE Healthcare). The eluates were concentrated using an AmiconUltra 15 Centrifugal Filter Unit 10 kDa (Merck Millipore) and filtered through 0.2-μm filter. The final sample was stored in 50 mM Tris–HCl, pH 7.4 and 150 mM NaCl buffer, aliquoted, and stored at −80°C.

### 2.6 Vaccine Formulation

The purified *Pf*CyRPA was lipidated by chemical conjugation to 1,2-dipalmitoyl-sn-glycero-3-phosphoethanolamine-N-[4-(p-maleinimidomethyl) cyclohexane-carboxamide)] (N-MCC-DPPE, Corden Pharma, Liestal, Switzerland) after limited modification of lysine residues with 2-iminothiolane (Merck & Cie, Schaffhausen, Switzerland) to introduce free thiol groups. The lipidated *Pf*CyRPA antigens were subsequently inserted into the virosome lipid membrane, as previously described (Amacker et al., 2020). Virosomes were prepared by Mymetics from inactivated and purified influenza virus [A/Brisbane/59/2007 (H1N1), Seqirus, Australia] with membrane-integrated 3M-052 adjuvant as a TLR7/8 agonist (3M company, St. Paul, United States). The final liquid virosome-*Pf*CyRPA contained approximately 40 μg/mL of *Pf*CyRPA, 6 μg/mL of hemagglutinin (HA), and 15 μg/mL of 3M–052 adjuvant and was supplied in 50 mM HEPES buffer pH 7.4, 142.5 mM NaCl. Quality controls were conducted using ELISA methods for determining the concentration (μg/mL) of *Pf*CyRPA, HPLC for 3M-052, and single radial immunodiffusion assay (SRID) for HA concentration (μg/mL). Dynamic light scattering (DLS) for determining the virosome particle size and homogeneity (polydispersity index, PDI) was performed on a Malvern Zetasizer Nano S (Malvern Instruments, Worcestershire, United Kingdom). Each sample was analyzed in triplicate at 25°C, and each replicate was measured six times to obtain the average particle size.

### 2.7 Rabbit Immunization

New Zealand rabbits were immunized and bled at Kaneka Eurogentec S.A. (Belgium). The animals (*n* = 2) were given three subcutaneous immunizations (30 μg antigen per dose) in intervals of 4 weeks. Blood was collected before each immunization and 9 days after the final injection. Total serum IgG was purified from rabbit sera using protein A columns (Cytiva) and assessed for activity in a single-cycle *in vitro* GIA with the *Pf* 3D7 strain.

### 2.8 Analytics

#### 2.8.1 Cell Concentration and Viability

Cell counting was performed in a Cedex HiRes Analyzer (Roche), and viability was assessed using the trypan blue exclusion method.

#### 2.8.2 Baculovirus Titration

Baculovirus titers were determined using the MTT assay as described elsewhere (Mena et al., 2003; Roldão et al., 2009).

#### 2.8.3 SDS-PAGE and Western Blot

Western blot analysis was performed as reported elsewhere (Correia et al., 2020). For the *Pf*CyRPA identification, mouse monoclonal antibodies c12 anti-CyRPA (provided by Prof. Gerd Plushke from Swiss TPH, Switzerland) and anti-6xHis tag (Thermo Scientific) were used at dilutions of 1:3000 or 1:1000, respectively. As a secondary antibody, an anti-mouse IgG antibody conjugated with alkaline phosphatase was used at a dilution of 1:5000 (Sigma, Ref.: A3438). A relative *Pf*CyRPA protein titer (mg/L) was determined by densitometry analysis of the Western blot performed using FIJI software (Schindelin et al., 2012). The expected MW of *Pf*CyRPA is 43 kDa.

#### 2.8.4 Total Protein Concentration

Total protein concentration was determined by spectrophotometry at 280 nm on the mySPEC (VWR) and using the Micro BCA Protein Assay Kit (Thermo Fisher Scientific) following the manufacturer’s instructions.

#### 2.8.5 Size Exclusion Chromatography

Purified CyRPA protein was analyzed using an HPLC system equipped with Photodiode Array Detector (Waters). CyRPA protein samples were injected in an XBridge BEH 125 Å SEC 3.5-μm HPLC column (Waters) equilibrated in 0.1 M sodium phosphate with 0.2 M NaCl, pH 7.4. The system flow rate was maintained at 0.86 mL/min, and eluted proteins were detected at 280 nm. Then 20 mg of protein was injected in each HPLC run.

#### 2.8.6 Differential Scanning Fluorimetry

DSF was performed in a Quant Studio 7 Flex Real-Time PCR System (Thermo Fisher Scientific), with excitation and emission wavelengths of 580 and 623 nm, respectively, using a MicroAmp^TM^ EnduraPlate^TM^ Optical 96-Well Fast Clear Reaction Plate with Barcode (Thermo Fisher Scientific). The samples were heated from 25°C to 90°C with stepwise increments of 0.016°C per second, followed by the fluorescence read-out. For each well, 20 µL final volume with 2 µg of *Pf*CyRPA protein and 2-fold of ROX™ Passive Reference Dye (Thermo Fisher Scientific) was prepared with protein purification buffer. The assays were carried out in triplicates, and the results were analyzed in Protein Thermal Shift^TM^ Software V1.3.

#### 2.8.7 Particle Size and Homogeneity

Dynamic light scattering (DLS) was performed on a SpectroLight 600 (Xtal Concepts) and Zetasizer Nano S instrument (Malvern) to determine population homogeneity, based on the polydispersity index (PDI) of purified *Pf*CyRPA protein particles and virosome-*Pf*CyRPA population, respectively. All the samples were pipetted (1 µL per well) onto a 96-well Vapor Batch Plate (Jena Bioscience GmbH). Before usage, the plates were filled with paraffin oil (Cat N. 18512; Merck) to protect sample solutions from drying out. The laser wavelength was 660 nm at a power of 100 mW. The scattering angle for placement of the detector was fixed at 142°. All samples were measured at constant 20°C, one scan per drop with 20 measurements of 20 s each.

#### 2.8.8 Peptide Identification by LC-MS

Disulfide bonds in *Pf*CyRPA protein were identified by LC-MS. Briefly, the peptides derived from proteolysis under native conditions by the endoproteases LysC, AspN, and trypsin were analyzed by LC-MS (X500B QTOF, ABSciex). The protein samples (10 μg of purified *Pf*CyRPA) were digested with AspN, LysC, and trypsin overnight at 37°C, without reduction to maintain intact disulfide bonds. External calibration was performed using beta-galactosidase digest (ABSciex). The twelve most intense precursor ions from the MS spectra were selected for MS/MS analysis. The data were acquired in positive TOF-MS mode using an X500B QTOF with a Turbo V ion source (ABSciex) mass spectrometer connected to the ExionLC AD UPLC system. Peptides were separated on bioZen 2.6-μm peptide XB-C18 column (2.1 × 150 mm) by a 1–90% gradient of acetonitrile in water containing 0.1% formic acid at a flow rate of 200 μl/min over 64 min and eluted into the mass spectrometer. The raw MS and MS/MS data were analyzed using Explorer software of SCIEX OS-Q and the BioPharmaView software 3.0 (Sciex) for peptide identification using the sponsor protein sequence.

#### 2.8.9 Enzyme-Linked Immunosorbent Assay

For the analysis of the binding reactivity of anti-*Pf*CyRPA mAbs with purified *Pf*CyRPA, Nunc MaxiSorp^TM^ flat-bottomed 96-well ELISA plates (Thermo Fisher Scientific) were coated overnight at 4°C with 3 μg/mL of *Pf*CyRPA protein produced either in HEK293 or High Five cells. The wells were then blocked with 5% (w/v) milk powder in phosphate-buffered saline (PBS) for 1 h at room temperature, followed by washing three times with PBS containing 0.05% (v/v) Tween-20. The plates were then incubated with serial dilutions of mAbs in PBS for 1 hat room temperature. After washing, the plates were incubated with goat anti-mouse (Sigma) conjugated to horseradish peroxidase (HRP) secondary antibody (Sigma) for 1 h at room temperature. Tetramethylbenzidine (TMB) was used as the substrate (KPL). The reaction was stopped after the appropriate time with 0.5 M H_2_SO_4_, and the absorbance was read at 450 nm with the Sunrise absorbance plate reader (Tecan). Data were processed and analyzed using GraphPad Prism 8. *Pf*CyRPA concentration in the virosome lipid particles was determined by ELISA and native immunoblot. The final vaccine was prepared by dilution of the intermediate mixture to the required final antigen concentration for the animal study (2 g *Pf*CyRPA per dose) and prepared in HN buffer (50 mM HEPES pH 7.4, 142 mM NaCl), followed by a final filtration step on a 0.22-µm PVDF syringe filter.

#### 2.8.10 Single Radial Immunodiffusion Assay

Determination of the influenza hemagglutinin (HA) concentration in virosome-*Pf*CyRPA was performed by SRID at Confarma France SAS (Hombourg, France).

#### 2.8.11 *In Vitro* Growth Inhibition Assay

Total serum IgG was assessed for activity in a single-cycle *in vitro* GIA. Briefly, synchronized *P. falciparum* 3D7 trophozoites were adjusted to 0.5% parasitemia and then incubated for 48 h with various concentrations of purified rabbit IgG in PBS at 1% hematocrit. Each culture was set up in triplicate in 96-well flat-bottomed culture plates. The erythrocytes were then washed and resuspended in PBS supplemented with hydroethidine fluorescent vital stain (15 μg/mL) and incubated at room temperature for 45 min. After washing, the final parasitemia was quantified by flow cytometry in a BD FACSCalibur flow cytometer by BD CellQuest software. Pooled purified IgG from collected pre-immune sera of both animals was used as a negative control. Percent inhibition was calculated relative to infection control wells containing PBS only. Data were processed and analyzed using GraphPad Prism 8.

### 2.9 Statistically Designed Experiments

Culture supplements and temperature were selected as factors for the screening DoE. A full factorial design with two levels and five factors was used to evaluate the effect of each parameter and their interactions with each other (Supplementary Table S1). This design allowed the determination of the main effects and all two-factor interactions without co-founding. A total of nineteen experiments were performed with three duplicates of the center point. For optimization of DoE, a central composite circumscribed design composed of a full or fractional factorial design and star points was implemented for optimization of conditions selected from the screening DoE (Supplementary Table S2). A total of eleven experiments were performed with three duplicates of the center point. The response for both screening and optimization DoEs was defined as the fold improvement in the *Pf*CyRPA titer (assessed by densitometry analysis of Western blot images and for cell viabilities between 30 and 40%) of each experiment when compared to standard culture conditions (i.e., 27°C without supplementation). MODDE® 12.1 Pro software (Sartorius) was used to determine significant effects, interactions, and distribution of the data.

### 2.10 Statistical Analysis

Data were expressed as mean ± standard deviation. Differences were tested by one-way ANOVA with *post hoc* Tukey’s multiple comparison analysis methods and Dunnett’s multiple comparison test (adjusted *p*-value < 0.05 was considered statistically significant) and were tested by t-test unpaired Gaussian distribution (adjusted *p*-value < 0.05 was considered statistically significant).

### 2.11 Data Availability Statement

The sensitive nature of some of the reagents used in this study (e.g., cell lines, plasmids, baculoviruses, virosomes, and antibodies) means that they are only readily available internally to the author’s institutions staff for R&D purposes. For external researchers, approval of reagent requests may be obtained via email addressed to the corresponding author.

## 3 Results

### 3.1 *Pf*CyRPA Production in Insect Cells: *Sf-9* vs. High Five Cells

**FIGURE 1:**
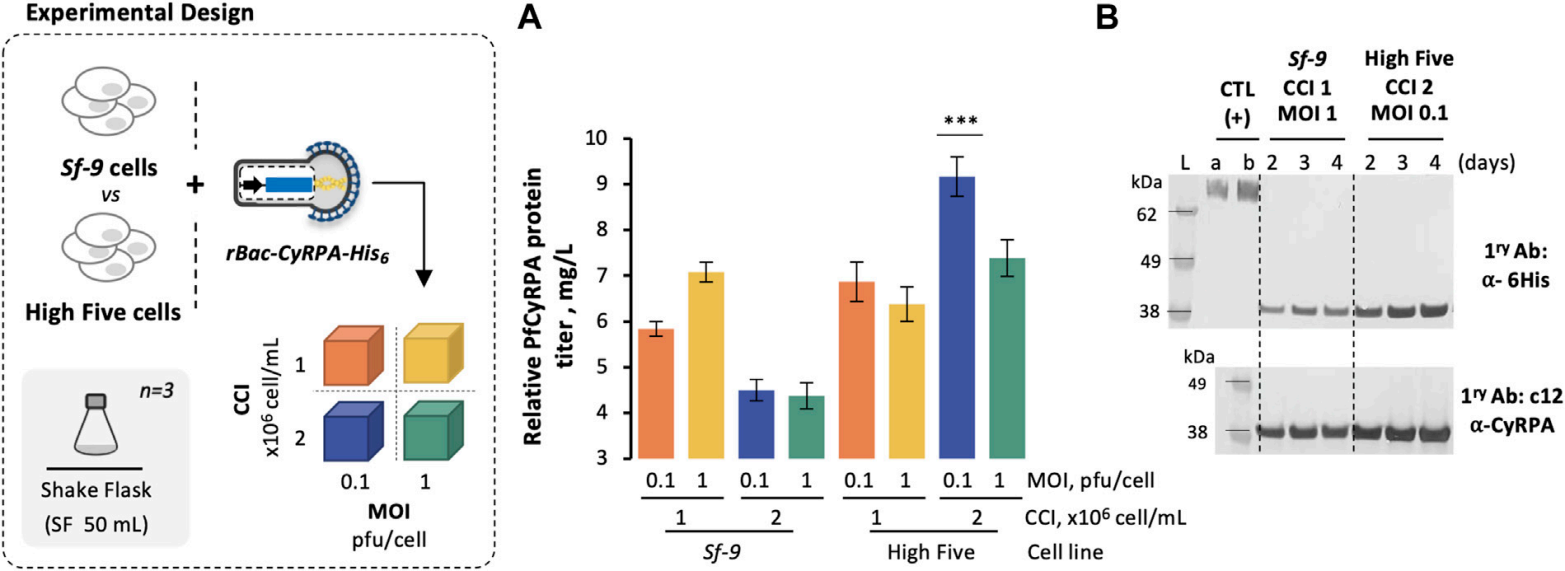
Production of *Pf*CyRPA using insect *Sf-9* and High Five cells. **(A)** Relative *Pf*CyRPA protein titer (mg/L), obtained by densitometry analysis of Western blot images. **(B)** Identification of *Pf*CyRPA in culture supernatant samples by Western blot. Positive control (CTL+) is an hsEGFR-His6 protein produced in-house at 50 and 75 ng; Ladder (L) is SeeBlue™ Plus2 Pre-stained Protein Standard. Data in the bar graph are expressed as mean ± standard deviation (relative to three biological replicates, *n* = 3). Statistical significance was tested by one-way ANOVA with Dunnett’s multiple comparison analysis methods, comparing the mean of each column to optimal condition (i.e., CCI = 2 × 10^6^ cell/mL and MOI = 0.1 pfu/cell, High Five cells); *** = adjusted *p*-value<0.001 was considered statistically significant.

*Sf9* and High Five cells were infected with rBac-CyRPA-His6 at different CCIs (1 and 2 × 10^6^ cell/mL) and MOIs (0.1 and 1 pfu/cell), and their growth and expression kinetics were assessed in small-scale shake flasks (SF) (Supplementary Figure S1 and Figure 1 **)**. The highest *Pf*CyRPA titer was achieved in High Five cells when infected at CCI = 2 × 10^6^ cell/mL with MOI = 1 pfu/cell; thus, this cell line and infection strategy were used for subsequent experiments.

### 3.2 Scale-Up *Pf*CyRPA Production: High Five vs. HEK293 Cells

The feasibility of producing *Pf*CyRPA in High Five cells was demonstrated in controlled, scalable 2-L stirred tank bioreactors (STB) and compared to HEK293 cells; small-scale SF was used to assess scalability.

**FIGURE 2:**
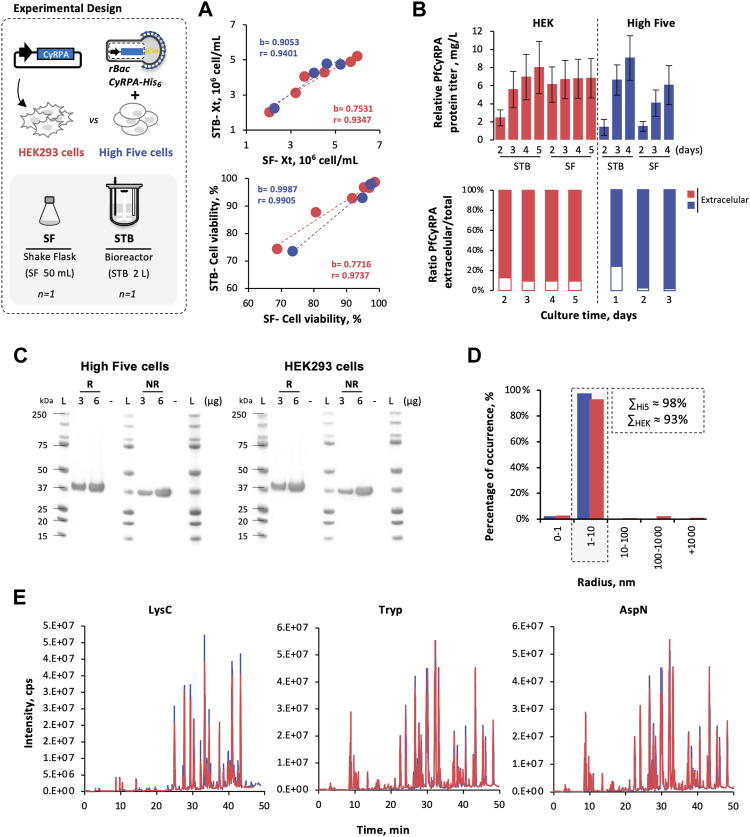
Scale-up *Pf*CyRPA production in insect and mammalian cells. **(A)** Cell growth and viability kinetics obtained in STB and SF cultures. **(B)** Relative *Pf*CyRPA protein titer (mg/L) and the ratio of extracellular/total *Pf*CyRPA (%), obtained by densitometry analysis of Western blot images. **(C)** SDS-PAGE of purified *Pf*CyRPA, under reduced (R) and non-reduced (NR) conditions; Ladder (L) is SeeBlue™ Plus2 Pre-stained Protein Standard. **(D)** Dynamic light scattering profile of purified *Pf*CyRPA. **(E)** Peptide identification of purified *Pf*CyRPA, digested with LysC, trypsin, and AspN, by LC-MS using X500B QTOF (ABSciex). Color code: High Five cells (blue) and HEK293 cells (red). Data in bar graphs are expressed as mean ± standard deviation (relative to three technical replicates, *n* = 3). Statistical significance was tested by t-test unpaired assuming Gaussian distribution.

Cell growth and viability kinetics in STB and SF were similar for both cells. With regression coefficients (b) and Pearson’s correlations (r) close to 1 (Figure 2A). Likewise, *Pf*CyRPA titers (estimated by densitometry analysis of Western blot images, Supplementary Figure S2), achieved in STB and SF, are comparable (Figure 2B). The intracellular *Pf*CyRPA content at the time of harvest was also assessed (only for STB runs), and results have shown that 91–97% of total *Pf*CyRPA produced was secreted, with no apparent difference between High Five and HEK293 cells.

High Five and HEK293-derived *Pf*CyRPA produced in STB were purified, and similar recovery yields were achieved (Table 2). Both proteins present purities above 90% (Figure 2C), similar melting temperatures (Tm, ≈ 61°C) (Table 2), and identical dimensions (Figure 2D). In addition, mass spectrometry analysis of proteolytic fragments from both *Pf*CyRPA proteins revealed similar sequence coverage and the same disulfide bond numbers (Figure 2E).

**TABLE 2:** Effect of expression and purification parameters on *Pf*CyRPA yield and protein characteristics.

Cells	Culture temperature	Culture medium supplementation	Affinity tag	Recovery yield	Calculated *Pf*CyRPA MW	Purity	Melting temperature	Final yield
	°C	Additives		(%)^b^	(Da)^a^	(%)^a^	(°C)	(mg/L)
HEK 293	27	—	6×–His	59	39829	> 90%	61.4 ± 0.3	21
High Five		—	6×–His	63	39829	> 90%	61.4 ± 0.3	15
		0.26 × antioxidants	6×–His	56	39818	> 90%	60.0 ± 1.3	21
		0.26 × antioxidants	4×–His	61	42458	> 90%	60.1 ± 1.6	26
		0.26 × antioxidants	C-tag	35	51094	> 90%	61.0 ± 1.3	13

a Determined by HPLC-SEC.

b Determined by densitometry analysis of Western blot for *Pf*CyRPA antibody.

The results obtained confirm the feasibility and scalability of producing *Pf*CyRPA in insect High Five cells.

### 3.3 Antigenicity and Immunogenicity of *Pf*CyRPA Produced Using Insect Cells

**FIGURE 3:**
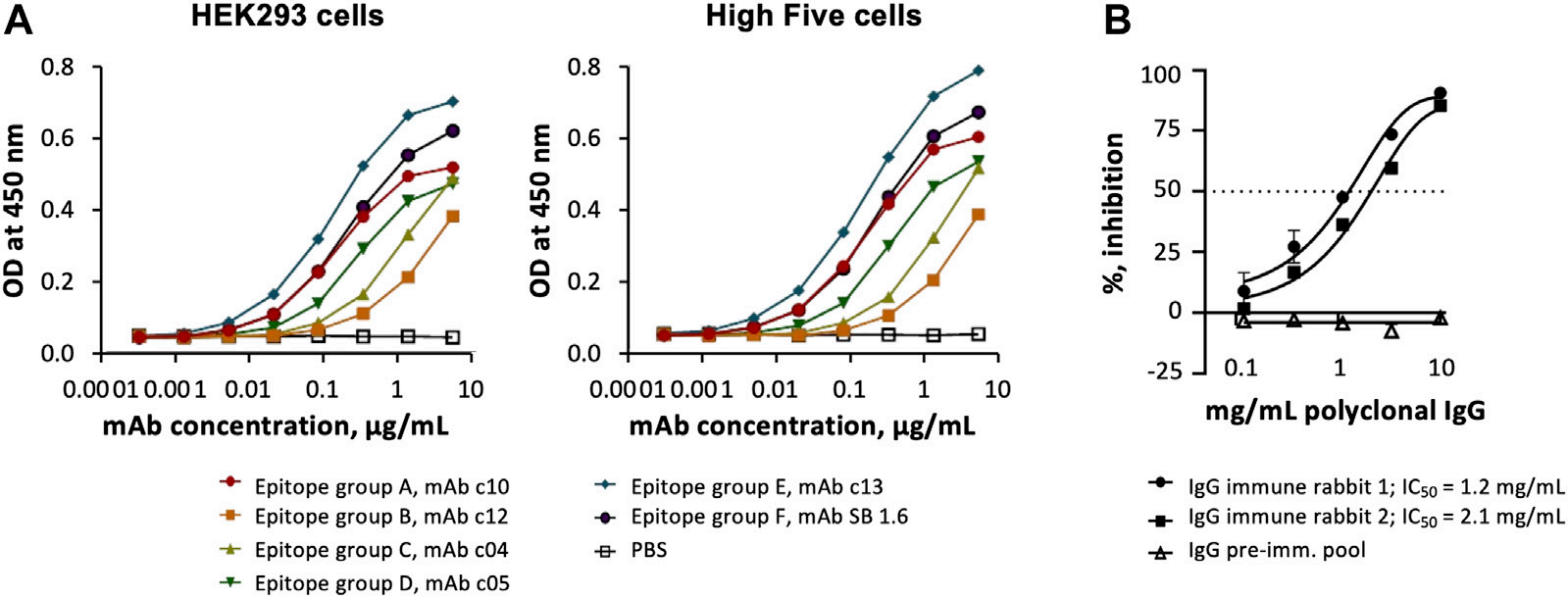
Antigenicity and immunogenicity of *Pf*CyRPA produced in insect and mammalian cells. **(A)** Binding of anti-*Pf*CyRPA mAbs recognizing different epitopes on purified *Pf*CyRPA protein by ELISA. **(B)** *In vitro* parasite growth-inhibitory activity of polyclonal IgG antibodies generated in rabbits upon immunization with High Five cells derived *Pf*CyRPA. Data are shown as mean ± standard deviation of triplicate wells of a single assay, and are representative of two independent runs. For each IgG preparation a four-parameter sigmoidal dose–response curve was fitted to the relationship between log_10_ (antibody concentration) and % inhibition and then used to interpolate IC_50_ values (antibody concentrations giving 50% growth inhibition).

Sets of anti-*Pf*CyRPA mAbs have been grouped into six epitope groups (Favuzza et al., 2017) by determining competition for antigen binding and reactivity patterns with overlapping fragments of *Pf*CyRPA as described elsewhere (Dreyer et al., 2012; Favuzza et al., 2016). No differences were observed, when the reactivity of representatives of these mAbs specific for the epitope groups A-F was tested with purified *Pf*CyRPA produced using insect or mammalian cells (Figure 3A). Noteworthy, the immunization of rabbits with a lipid-based particle formulation of purified *Pf*CyRPA expressed using High Five cells led to the generation of antibody responses that prevented the multiplication of *P. falciparum* in a parasite GIA (Figure 3B **)**.

### 3.4 Optimizing *Pf*CyRPA Production in High Five Cells: Design of Experiments

The impact of culture temperature and medium supplementation on *Pf*CyRPA expression was assessed in small-scale SF using ) a full factorial DoE to evaluate the effect of each parameter and their interaction with each other (screening DoE), followed by ) a central composite DoE for optimization of the previous conditions selected (optimization DoE). The culture temperatures explored were 21, 27, 31.5, and 36 C. The culture medium supplements evaluated were lipids, polyamines, antioxidants, and α-ketoglutarate (Table 1). The cultures at 27°C without supplements were used as the control.

**FIGURE 4:**
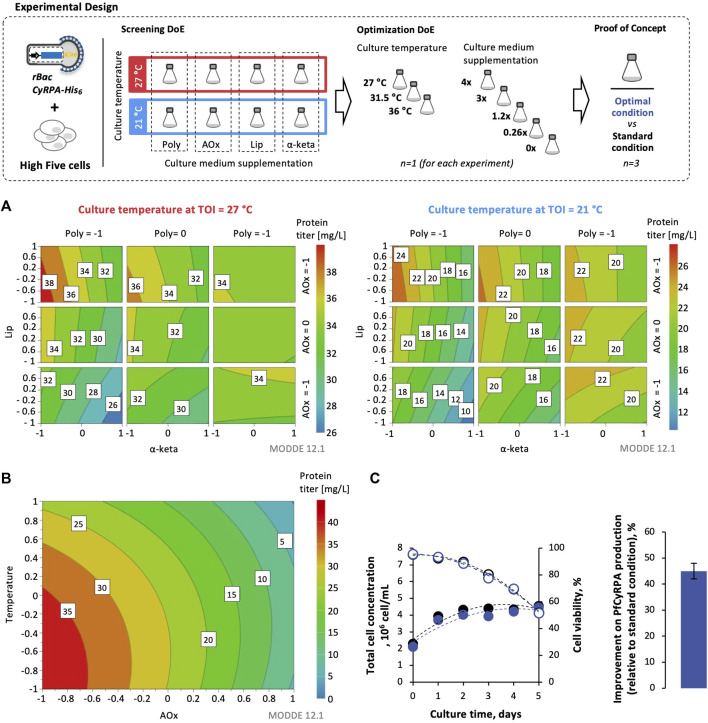
Design of experiments (DoE) for optimizing *Pf*CyRPA production in High Five cells. Response surface models for *Pf*CyRPA protein titer (mg/L) from screening experiments **(A)** and optimization experiments **(B)**. **(C)** Cell growth and viability kinetics in optimal conditions identified by DoE. Improvement of *Pf*CyRPA protein titer (mg/L) in optimal conditions identified by DoE vs. standard condition (i.e., cultures at 27°C and no supplementation). DoE images were obtained using MODDE® 12.1 Pro software (Sartorius). Data in bar graphs are expressed as mean ± standard deviation (relative to three technical replicates, *n* = 3). Abbreviations: TOI, time of infection; Lip, lipids; Poly, polyamines; α-keta, α-ketoglutarate; AOx, antioxidants.

Supplementation of antioxidants at the time of infection to cultures at 27°C (Supplementary Table S1) enhanced *Pf*CyRPA expression when compared to control cultures (Figure 4A—Western blot images of these experiments are reported in Supplementary Figure S3A), and thus, antioxidants were selected for further optimization experiments.

A central composite experimental design was used to optimize the concentration of antioxidants and the culture temperature. The experimental design matrix is shown in Supplementary Table S2. Based on the adjusted response surface model, it was possible to predict the best condition to maximize *Pf*CyRPA expression: cultures at 27°C and supplemented with 0.26 × antioxidants at the time of infection (Figure 4B–Western blot images of these experiments are reported in Supplementary Figure S3B). This optimal condition was tested in small-scale SF, with an approximately 45% increase in *Pf*CyRPA titer observed when compared to the control culture (Figure 4C, Western blot images of these experiments are reported in Supplementary Figure S4).

### 3.5 Optimizing *Pf*CyRPA Purification in High Five Cells: Exploring Different Affinity Tags

The impact of different affinity tags (i.e., 6×-His, 4×-His, and C-tag) on *Pf*CyRPA recovery yields was assessed in controlled, scalable 2-L STB. The optimal culture conditions identified before were used: CCI = 2 × 10^6^ cell/mL, MOI = 0.1 pfu/cell, culture temperature of 27°C, and culture supplementation with 0.26 x antioxidants.

**FIGURE 5:**
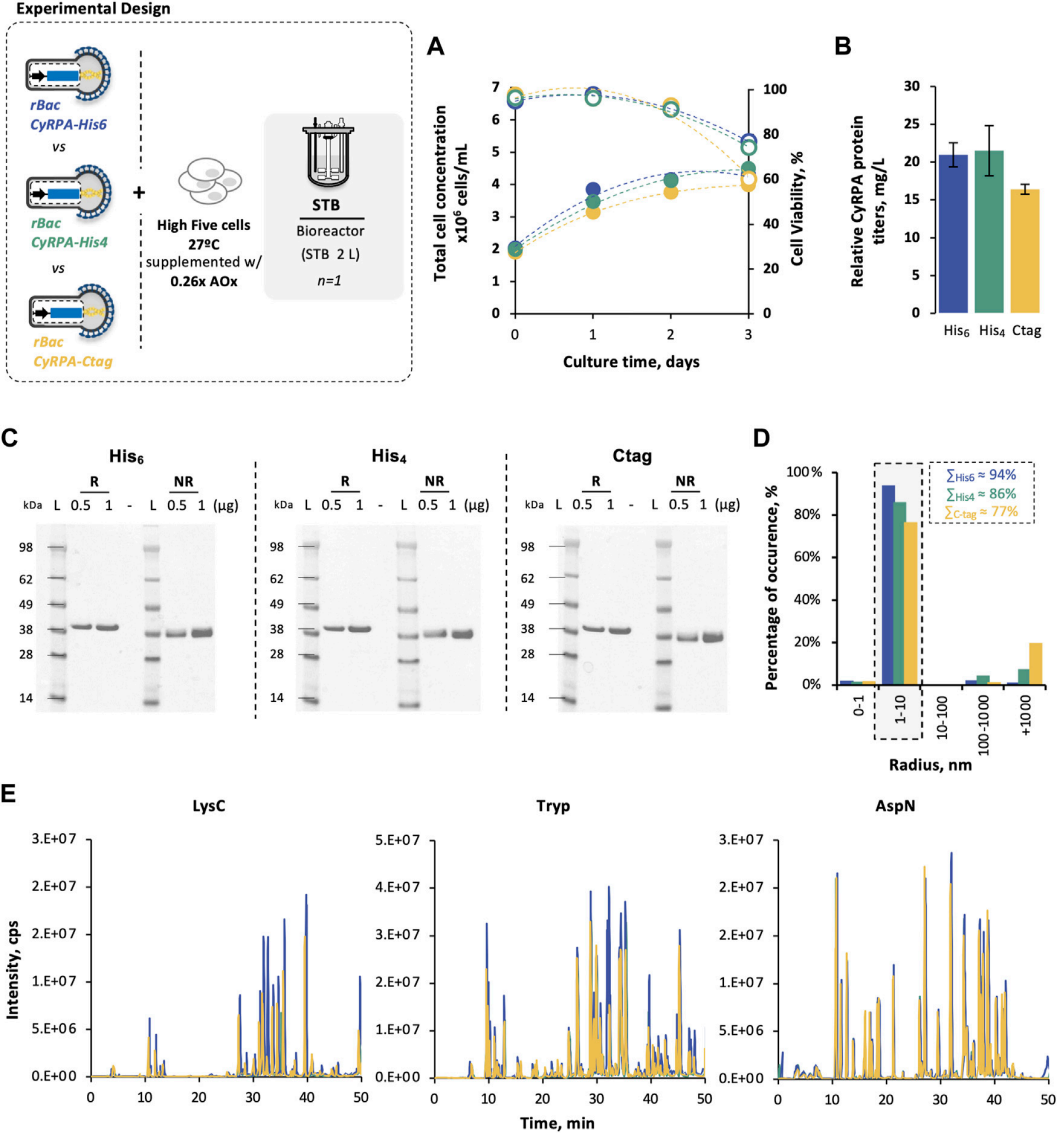
Impact of affinity tags on *Pf*CyRPA production in High Five cells. **(A)** Cell growth and viability kinetics obtained in STB cultures. **(B)** *Pf*CyRPA final yield (mg/L) after purification. **(C)** SDS-PAGE of purified *Pf*CyRPA, under reduced (R) and non-reduced (NR) conditions; Ladder (L) is SeeBlue™ Plus2 Pre-stained Protein Standard. **(D)** Dynamic light scattering profile of purified *Pf*CyRPA. **(E)** Peptide identification of purified *Pf*CyRPA, digested with LysC, trypsin, and AspN, by LC-MS using X500B QTOF (ABSciex). Color code: *Pf*CyRPA-His6 (blue); *Pf*CyRPA-His4 (green); *Pf*CyRPA-Ctag (yellow). Data in bar graphs are expressed as mean ± standard deviation (relative to three technical replicates, *n* = 3). Abbreviations: AOx, antioxidant.

Cell growth and viability kinetics were similar in all STB production runs (Figure 5A), with no apparent impact of affinity tag on *Pf*CyRPA expression (Supplementary Figure S4). Noteworthy, *Pf*CyRPA recovery yields were higher when using histidine tags than C-tag, with 4×-His slightly outperforming 6×-His (Table 2), thus resulting in a higher final yield (Figure 5B). Independently of the affinity tag used, purified *Pf*CyRPA proteins showed high purity (Figure 5C), similar melting temperatures (Table 2) and size (Figure 5D), and comparable sequence coverage with the same disulfide bonds detected (Figure 5E).

These results confirm the feasibility of the strategy herein proposed (i.e. insect High Five cells cultured at 27°C with supplementation with 0.26 × antioxidants at time of infection, and 4×-His affinity tag) for the production of *Pf*CyRPA protein.

## 4 Discussion

To date, the production of *Pf*CyRPA antigen has been only attempted in human (HEK293) and bacteria (*E. coli*) cells. The high demand for a malaria vaccine urges the development of production systems capable of providing high yield and high quality (e.g., complex posttranslational modifications) at low cost and with straightforward scalability. In this study, two insect cell lines were compared for *Pf*CyRPA expression, with results indicating that High Five cells can result in higher volumetric titers than *Sf-9* cells. When compared to human HEK293 cells, the insect High Five cells induced similar *Pf*CyRPA expression levels in shorter time frames (i.e., 3 vs. 5 days); the insect cells also usually allow reduced manufacturing costs (Rhodes, 1996; Kost et al., 2005; Yee et al., 2018). Importantly, yields achieved are within those reported in the literature, that is, 18 mg/L using HEK293 cells (Favuzza et al., 2016). Purified insect-derived *Pf*CyRPA presents similar purity, protein stability, conformation, and sequence coverage to its human-derived counterpart. In addition, *Pf*CyRPA produced in both hosts showed no differences in reactivity to the specific mAbs for different epitope groups, confirming the quality of the *Pf*CyRPA produced using High Five cells. Noteworthy, a lipid-based virosome particle formulation with membrane-anchored *Pf*CyRPA expressed in High Five cells resulted in the generation of antibody responses in rabbits that efficiently impeded the multiplication of *P. falciparum* in a parasite growth *in vitro* inhibition assay, with an IC_50_ in the range of 1–2 mg/mL of polyclonal serum. Similar outcomes have been reported for other malaria vaccine candidates, particularly with the conserved *Pf*RH5 antigen produced in insect and mammalian cells (Bustamante et al., 2013; Patel et al., 2013), leading also to comparable *in vitro* growth inhibition. Taken together, these results demonstrate that recombinant *Pf*CyRPA protein expressed with the IC-BEVS (in High Five cells) represents an improved blood-stage candidate antigen for inclusion in a broadly cross-reactive malaria vaccine.

A DoE approach was used to optimize the expression of *Pf*CyRPA in High Five cells by manipulating two factors: 1) culture temperature and 2) culture medium supplementation. Best result was achieved at standard culture temperature (27°C) by supplementing the culture medium with (0.26×) antioxidants at the time of infection, improving *Pf*CyRPA volumetric titer by 50% when compared to control cultures. Culture medium supplementation strategies using antioxidants have shown to increase cell-specific yields of infectious baculovirus particles by reducing the oxidative cellular microenvironment induced by baculovirus infection, further resulting in increased protein expression (Monteiro et al., 2016). Interestingly, combining different culture medium supplements (e.g., antioxidants with polyamines) with or without culture temperature shifts did not improve *Pf*CyRPA expression as previously reported in other studies for similar biological entities (Aucoin et al., 2007; Carinhas et al., 2010; Monteiro et al., 2016; Somasundaram et al., 2016).

Although affinity tags can improve protein recovery yields in purification, they can also interfere with protein’s physicochemical properties, functionality, and immunogenicity (Arnau et al., 2006). In this study, three different affinity tags (6×-His, 4×-His, and C-tag) were explored as to their potential to improve *Pf*CyRPA recovery yields. Independently of the affinity tag used, *Pf*CyRPA could be detected in the culture supernatant as early as 24 h postinfection. Importantly, *Pf*CyRPA recovery yields were higher when using histidine tags than when using C-tag, with the 4×-His process outperforming 6×-His in terms of final yields achieved (26 vs. 21 mg/L). The preference of one affinity tag over another depends on the properties of the protein of interest, with some studies reporting the same outcome as that herein obtained and others showing the opposite (Jin et al., 2017). Noteworthy, the recovery yields achieved in our study with histidine tags are more than those reported for other malaria vaccine targets using the same affinity tag (Hjerrild et al., 2016). Comparing the 4× vs. 6×-His-tag, the use of longer histidine tags has a positive impact on recovery yields, enabling more stringent and efficient washing steps. On the other hand, shorter histidine tags minimize potential interferences on protein biochemical properties (Bornhorst & Falke, 2000). Despite their differences, the three affinity tags explored in this study did not impact protein quality or stability. Taken together, these results suggest that 4×-His would be the ideal affinity tag to use for scale-up and clinical biomanufacturing of *Pf*CyRPA antigen.

## 5 Conclusion

This work demonstrates the feasibility and scalability of producing *Pf*CyRPA, a promising blood-stage candidate malaria antigen for inclusion into a broadly *Pf* cross-strain reactive malaria vaccine, using insect High Five cells with BEVS. We describe for the first time the use of insect cells for *Pf*CyRPA production in combination with different upstream and downstream bioprocess optimization strategies to improve protein expression and purification. IC-BEVS is a scalable system yielding high protein titers in short time frames, herein employed to produce a correctly folded *Pf*CyRPA protein capable of eliciting neutralizing antibodies in rabbits that inhibited multiplication of *P. falciparum.*

The presented work encourages not only the use of IC-BEVS as a robust platform for *Pf*CyRPA production but also the use of High Five cell-derived *Pf*CyRPA protein as an antigen candidate qualified for the inclusion in malaria vaccine.

## Data Availability

The raw data supporting the conclusion of this article will be made available by the authors, without undue reservation.
