# Supplementary figures and images for: Scalable Process for High-Yield Production of PfCyRPA Using Insect Cells for Inclusion in a Malaria Virosome-Based Vaccine Candidate
Source: Front Bioeng Biotechnol. 2022 May 20;10:879078. doi: 10.3389/fbioe.2022.879078 (PMC9163744; doi:10.3389/fbioe.2022.879078)

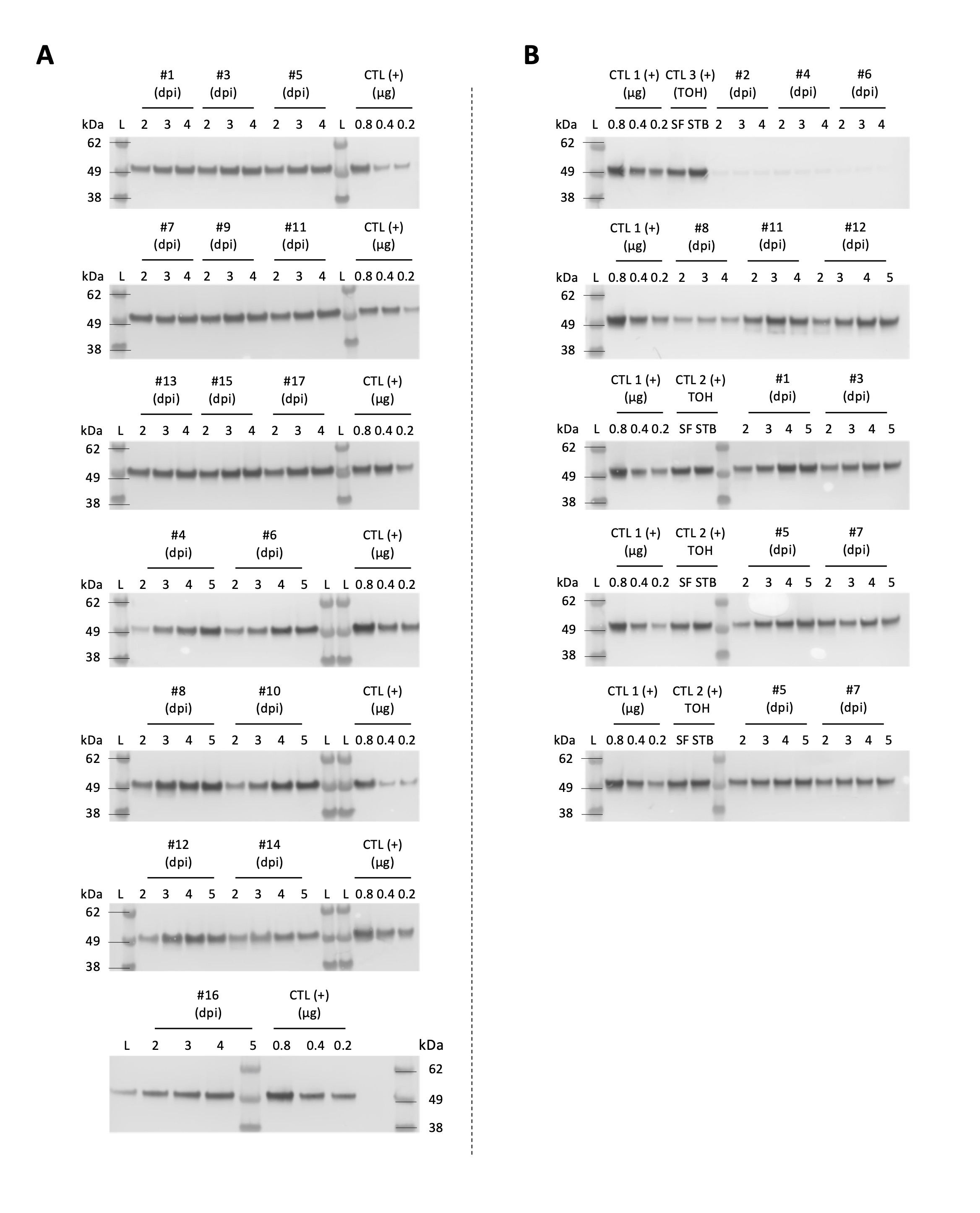

Supplement: Supplementary file 1 [file Image3.JPEG]

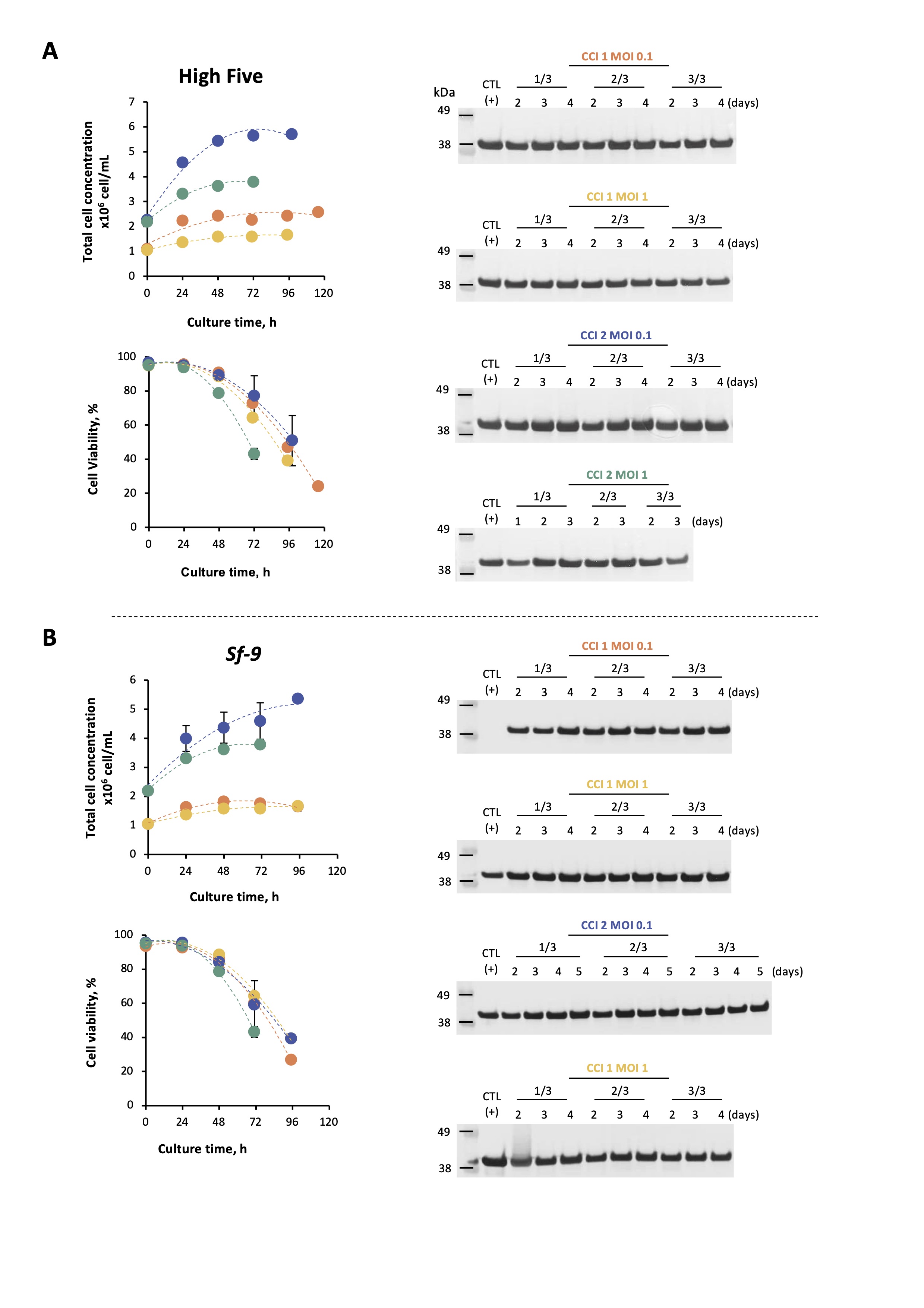

Supplement: Supplementary file 3 [file Image1.JPEG]

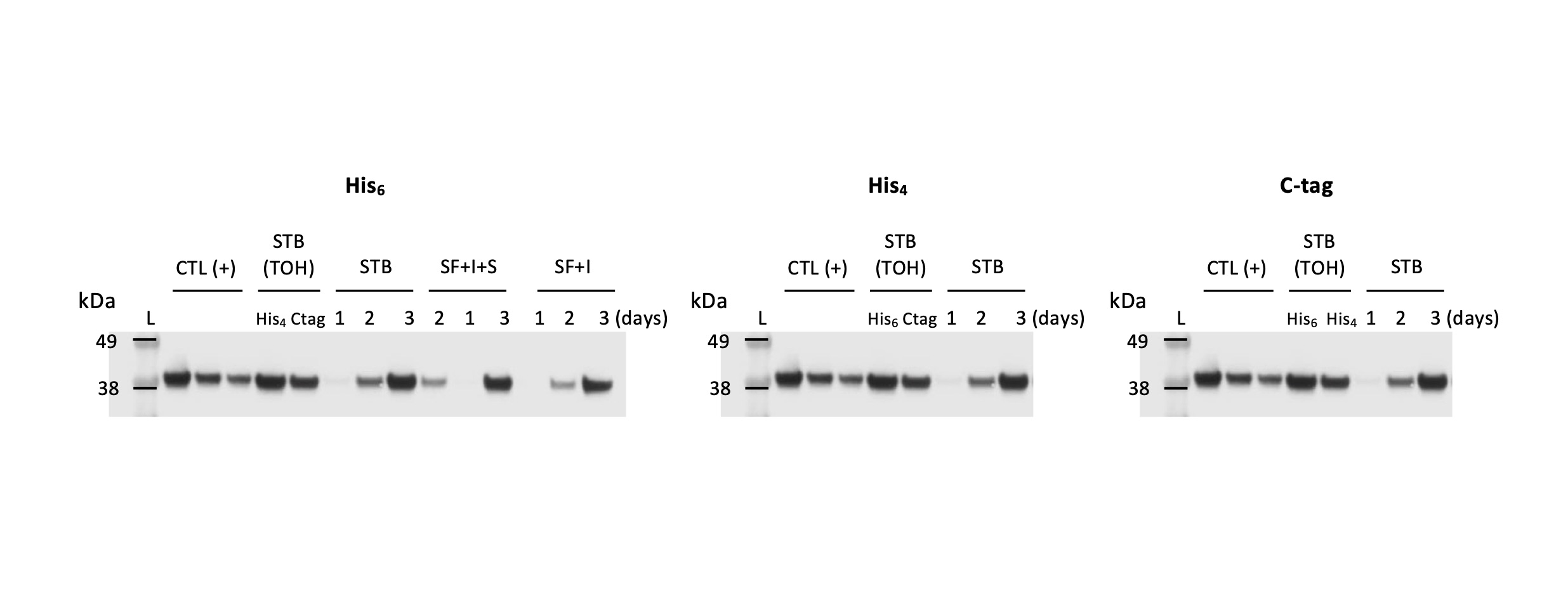

Supplement: Supplementary file 4 [file Image4.JPEG]

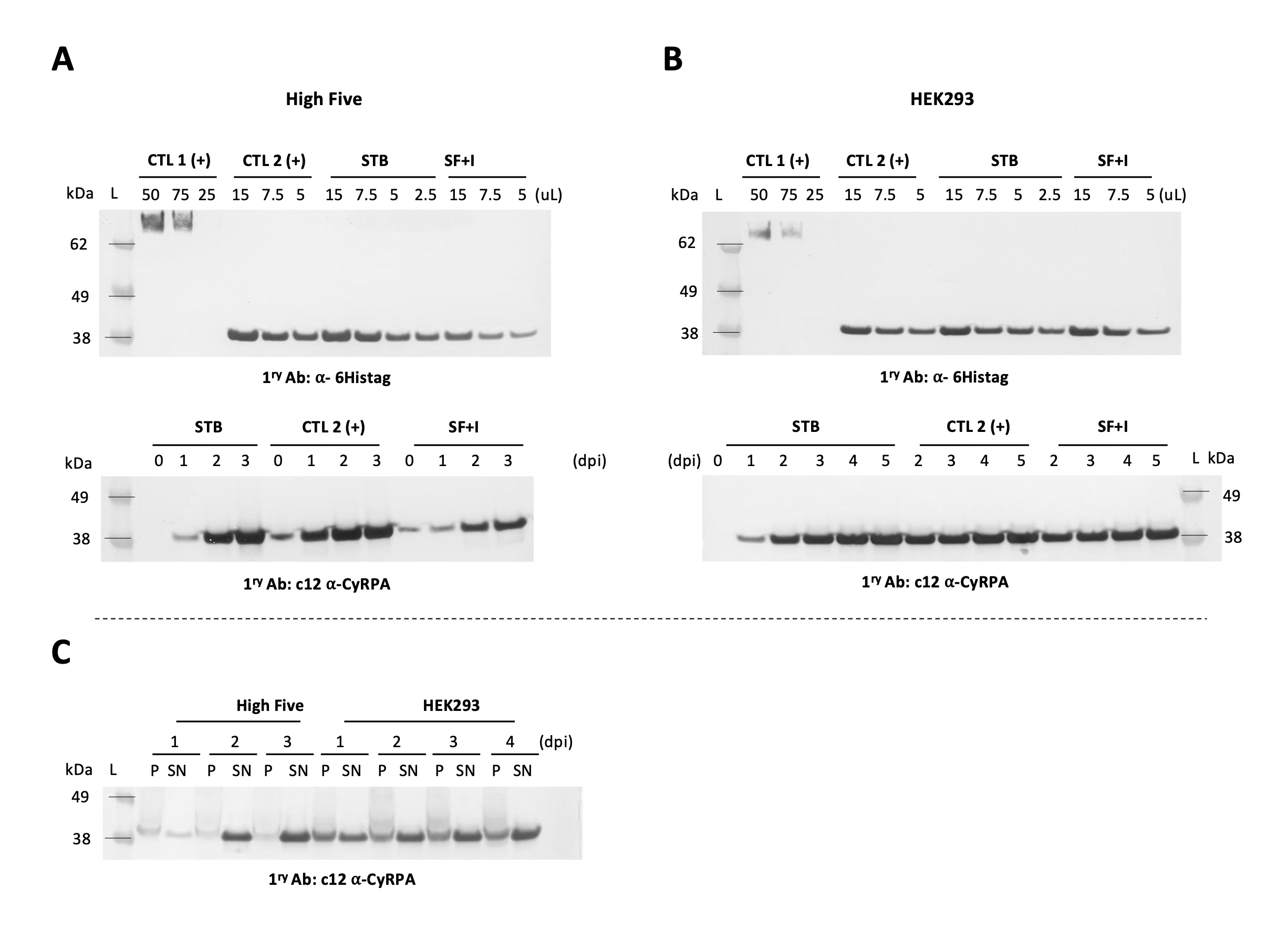

Supplement: Supplementary file 5 [file Image2.JPEG]
