# Supplementary material for: Scalable Process for High-Yield Production of PfCyRPA Using Insect Cells for Inclusion in a Malaria Virosome-Based Vaccine Candidate
Source: Front Bioeng Biotechnol. 2022 May 20;10:879078. doi: 10.3389/fbioe.2022.879078 (PMC9163744; doi:10.3389/fbioe.2022.879078)
Supplement: Supplementary file 2 [file Table1.DOCX]

**Tables**

**Table 1.** List of culture medium supplements.

| **Supplement** | **Abbreviation** | **Stock concentration** | | **Concentration added ^a^** | | **Supplier** | | **Reference** | |  |
| --- | --- | --- | --- | --- | --- | --- | --- | --- | --- | --- |
| Antioxidants | AOx | | 1000 x | | 1 x | | Sigma | | A1345 | |
| Polyamines | Pol | | 100 x | | 1 x | | Sigma | | G1404 | |
| Lipids | Lip | | 100 x | | 1 x | | Gibco | | 11905-031 | |
| Disodium α-ketoglutarate | α-k | | - | | 12 mM | | Sial | | K-3752 | |

^a^ *Concentration of supplements added at time of infection*

**Table 2.** Effect of expression and purification parameters on *Pf*CyRPA yield and protein characteristics

| ***Cells*** | ***Culture temperature*** | ***Culture medium supplementation*** | ***Affinity tag*** | ***Recovery yield*** | ***Calculated PfCyRPA MW*** | ***Purity*** | ***Melting temperature*** | ***Final Yield*** |
| --- | --- | --- | --- | --- | --- | --- | --- | --- |
|  | *°C* | *Additives* |  | *(%) ^b^* | *(Da) ^a^* | ***(%)*** *^a^* | *(ºC)* | *(mg/L)* |
| *HEK 293* | *27* | *-* | *6x - His* | *59* | *39829* | *> 90%* | *61.4 ± 0.3* | *21* |
| *High Five* |  | *-* | *6x - His* | *63* | *39829* | *> 90%* | *61.4 ± 0.3* | *15* |
|  |  | *0.26 x antioxidants* | *6x - His* | *56* | *39818* | *> 90%* | *60.0 ± 1.3* | *21* |
|  |  | *0.26 x antioxidants* | *4x - His* | *61* | *42458* | *> 90%* | *60.1 ± 1.6* | *26* |
|  |  | *0.26 x antioxidants* | *C-tag* | *35* | *51094* | *> 90%* | *61.0 ± 1.3* | *13* |

^a^ *Determined by HPLC-SEC*

*^b^ Determined by densitometry analysis of western blot for PfCyRPA antibody*

**Supplementary Tables**

**Table S1.**  Matrix design and response for screening DoE using a full factorial design with two levels and 5 factors.

| **Experiment #** | **Factors** | | | | | **Response** |
| --- | --- | --- | --- | --- | --- | --- |
|  | Culture temperature | Culture medium supplementation | | | | **Fold improvement in *Pf*CyRPA titer ^a^** |
|  |  | Lipids | Polyamines | Antioxidants | Alpha-ketaglutarate |  |
| 1 | -1 | -1 | -1 | -1 | 1 | 0.68 |
| 2 | 1 | -1 | -1 | -1 | -1 | 0.49 |
| 3 | -1 | 1 | -1 | -1 | -1 | 0.94 |
| 4 | 1 | 1 | -1 | -1 | 1 | 0.38 |
| 5 | -1 | -1 | 1 | -1 | -1 | 0.91 |
| 6 | 1 | -1 | 1 | -1 | 1 | 0.56 |
| 7 | -1 | 1 | 1 | -1 | 1 | 1.08 |
| 8 | 1 | 1 | 1 | -1 | -1 | 0.73 |
| 9 | -1 | -1 | -1 | 1 | -1 | 1.14 |
| 10 | 1 | -1 | -1 | 1 | 1 | 0.45 |
| 11 | -1 | 1 | -1 | 1 | 1 | 0.93 |
| 12 | 1 | 1 | -1 | 1 | -1 | 0.77 |
| 13 | -1 | -1 | 1 | 1 | 1 | 0.99 |
| 14 | 1 | -1 | 1 | 1 | -1 | 0.73 |
| 15 | -1 | 1 | 1 | 1 | -1 | 1.03 |
| 16 | 1 | 1 | 1 | 1 | 1 | 0.50 |
| 17 | -1 | -1 | -1 | -1 | -1 | 1 |
| 18 | -1 | -1 | -1 | -1 | -1 | 1 |
| 19 | -1 | -1 | -1 | -1 | -1 | 1 |

^a^ Relative to standard culture condition, i.e. 27 ºC without supplementation (average of experiments #17, #18 and #19)

*Table S1 footnote: All variables were studied at 2 levels: a low level coded as -1 and a high level coded as 1. For temperature -1 denote the 27 ºC and 1 denote the 21 ºC at time of infection; For lipids, antioxidants and polyamines -1 denote to without supplementation and 1 denote to supplementation with 1x of supplement; For α-ketoglutarate 1 denote to without supplementation and 1 denote to supplementation with 12 mM of supplement. Centre point: experiments 17, 18 and 19. Response is fold improvement in PfCyRPA titer relative to control condition (27 ºC without supplementation).*

**Table S2.**  Matrix design and response for optimization DoE using a central composite

circumscribed design composed of a full or fractional factorial design and star points.

| **Experiment #** | **Factors** | | **Response** |
| --- | --- | --- | --- |
|  | Culture temperature | Culture medium supplementation | **Fold improvement in *Pf*CyRPA titer ^a^** |
|  |  | Antioxidants |  |
| 1 | -1 | -1 | 1.4 |
| 2 | 1 | -1 | - |
| 3 | -1 | 1 | 1 |
| 4 | 1 | 1 | - |
| 5 | -1.414 | 0 | 1.2 |
| 6 | 1.414 | 0 | - |
| 7 | 0 | -1.414 | 1 |
| 8 | 0 | 1.414 | 0.2 |
| 9 | 0 | 0 | 0.9 |
| 10 | 0 | 0 | 0.5 |
| 11 | 0 | 0 | 1 |

^a^ Relative to standard culture condition, i.e. 27 ºC without supplementation (experiments # 9, #10 and #11)

*Table S2 footnote: All variables were studied at 5 levels: -1.414, -1, 0, 1 and 1.414; For temperature -1.414 and -1 denote to 27 ºC, 0 denote to 31.5 ºC and 1 and 1.414 denote to 36 ºC at time of infection; For antioxidants -1.414 denote to without supplementation, -1 denote to supplementation with 0.26 x of antioxidants; 0 denote to supplementation with 1.2 x of antioxidants, 1 denote to supplementation with 3x of antioxidants and 1.414 denote to supplementation with 4 x of antioxidants. Centre point: experiments 9, 10 and 11. Response is fold improvement in PfCyRPA titer relative to control condition (27 ºC without supplementation).*
